# Petunia as a model for MYB transcription factor action under salt stress

**DOI:** 10.3389/fpls.2023.1286547

**Published:** 2023-12-08

**Authors:** Baltasar Zepeda, Leo F. M. Marcelis, Elias Kaiser, Julian C. Verdonk

**Affiliations:** Horticulture and Product Physiology, Department of Plant Sciences, Wageningen University & Research, Wageningen, Netherlands

**Keywords:** MYB, transcription factors, salinity, petunia, quality traits

## Abstract

Salinity is a current and growing problem, affecting crops worldwide by reducing yields and product quality. Plants have different mechanisms to adapt to salinity; some crops are highly studied, and their salinity tolerance mechanisms are widely known. However, there are other crops with commercial importance that still need characterization of their molecular mechanisms. Usually, transcription factors are in charge of the regulation of complex processes such as the response to salinity. MYB-TFs are a family of transcription factors that regulate various processes in plant development, and both central and specialized metabolism. MYB-TFs have been studied extensively as mediators of specialized metabolism, and some are master regulators. The influence of MYB-TFs on highly orchestrated mechanisms, such as salinity tolerance, is an attractive research target. The versatility of petunia as a model species has allowed for advances to be made in multiple fields: metabolomic pathways, quality traits, stress resistance, and signal transduction. It has the potential to be the link between horticultural crops and lab models, making it useful in translating discoveries related to the MYB-TF pathways into other crops. We present a phylogenetic tree made with *Petunia axillaris* and *Petunia inflata* R2R3-MYB subfamily sequences, which could be used to find functional conservation between different species. This work could set the foundations to improve salinity resistance in other commercial crops in later studies.

## Effects of salinity on crops

Salinity is among the most detrimental factors affecting plant growth and crop yield. Currently, 20% of irrigated crop land is affected by salinity, and due to climate change this problem is expected to worsen (Li et al., 2023). Salt stress starts with osmotic stress, a rapid response that results in reduced water uptake by the roots, and is followed by an ionic stress, which occurs over a period of days to weeks (Munns and Tester, 2008). Osmotic stress reduces water uptake through the roots, with the first and most prominent effect being stomatal closure, which subsequently reduces the rates of CO_2_ assimilation and transpiration (Munns and Tester, 2008). Another consequence of osmotic stress is a reduction in the rates of cell elongation, hampering cell division and slowing down growth rates (Munns et al., 1995). Ionic stress is caused by a prolonged exposure to salinity, wherein sodium (Na^+^) and chloride (Cl^-^) accumulate in plant cells and cause, among other effects, an increased rate of leaf senescence (Rodríguez Coca et al., 2023). Due to leaf senescence and reduced leaf area expansion (caused by osmotic stress), plants may have a reduction in whole-plant light interception, and additionally leaves may show reduced photosynthetic capacity; both consequences directly impact biomass (Munns et al., 1995; Zörb et al., 2019). In most cases, yield is reduced when crops are grown under high salinity conditions (Zörb et al., 2019). In the case of fruits and vegetables, this could result in smaller final products or products that do not meet consumer quality standards (Saied et al., 2005). However, examples do exist where quality related metabolites are elevated under salinity stress, often leading to higher customer appreciation (Rouphael et al., 2018; Moles et al., 2019). Breeding for high productivity under salinity requires an understanding of different mechanisms that plants have evolved in their response to salinity.

## Molecular mechanisms of salt tolerance

Salt tolerance in plants involves intricate molecular mechanisms that aim to keep plants functioning. Higher Na^+^ ion concentrations inside plant cells act as competitive inhibitors of enzymes and other ions, such as potassium (K^+^), disrupt photosynthesis and cell homeostasis, and result in higher levels of Reactive Oxygen Species (ROS) (Zörb et al., 2019). Because these effects are detrimental for plant functioning, plants have developed several responses as adaptation mechanisms to soil salinity—such as osmotic adjustment, ion compartmentalization, and selective ion exclusion (Serrano and Rodriguez-Navarro, 2001; Parida and Das, 2005; Zhao et al., 2020). Several antiporters have been proposed as primary actors, to some extent, of the previously mentioned salinity stress resistance processes. These antiporters include the CHX, KEA, and NHX families, which play a critical role in the adaptation to stress of Na^+^ either by controlling the uptake into the membranes or by compartmentalizing into the vacuoles (Munns, 2005; Asif et al., 2011; Sze and Chanroj, 2018). To synchronize their expression in the tissues of interest, signal transduction depends on large, orchestrated changes in gene expression that can be induced by transcriptional modules (Mantri et al., 2007; Shaar-Moshe et al., 2017; Franzoni et al., 2019). In this light, we propose to investigate the involvement of a ubiquitous transcription factor (TF) family that may coordinate different physiological processes, and which in some cases have been shown to enhance salinity resistance (see below).

## What are MYB-TFs?

MYB-TFs were originally named after the avian myeloblastosis virus (MYB), and are among the most prominent TF families in plants (Klempnauer et al., 1982). They have been demonstrated to be a cornerstone of plant functioning (Allan and Espley, 2018; Wang X. et al., 2021). As an example, MYB-TFs are involved in the regulation of different central metabolism processes, such as cell fate specification in the root epidermis (Ryu et al., 2005), differentiation of cells into trichomes (Oppenheimer et al., 1991), pigmentation of flowers and tissues (Koes et al., 2005; Liu et al., 2021), and controlling stomatal aperture in guard cells (Cominelli et al., 2005; Liang et al., 2005).

Different classifications of MYB-TFs, depending on homology, have been described (Stracke et al., 2001; Dubos et al., 2010; Allan and Espley, 2018; Pucker et al., 2020). The structure of a MYB-TF consists of a highly conserved DNA binding domain (MYB domain) with a helix-turn-helix (HTH) motif. These HTH motifs form a DNA recognition site (R), allowing the interaction with different target DNA sequences (Dubos et al., 2010). Depending on the number of R sites, the MYB-TF family is subdivided into four subfamilies: 1R-MYB, R2R3-MYB, 3R-MYB, and 4R-MYB (Dubos et al., 2010). Among the four subfamilies of MYB-TFs, the R2R3-MYB subfamily is by far the largest, the best studied (see below, Du et al., 2012). Therefore, we focus on the R2R3-MYB subfamily and its potential interaction on salinity tolerance mechanisms.

## Why study MYB-TFs?

Several R2R3-MYBs have been characterized as master regulators, i.e., genes at the top of the hierarchy of a regulatory pathway (Kin Chan, 2013). MYB-TFs are an integral part of plant development, cell fate, and specialized metabolism. In particular for specialized metabolism, they have a key role as master regulators in processes related to anthocyanin biosynthesis (Quattrocchio et al., 2006; Pérez-Díaz et al., 2016; Feng et al., 2018; Zhou et al., 2019), fragrance (Verdonk et al., 2005; Yoshida et al., 2018), and lignin and secondary cell wall biosynthesis (Zhong et al., 2008). Even with the discovered functions, many other gene copies and different species remain uncharacterized, leaving many MYB-TFs with unknown roles.

The function of MYB-TFs is closely associated with different abiotic stress responses. For example, MYB-TFs were shown to be involved in drought, salt, and cold stress responses in wheat (*Triticum aestivum*), *Arabidopsis thaliana*, and rice (*Oryza sativa*) (Jung et al., 2008; Cai et al., 2011; Qu et al., 2022). *OsMYB91* coordinates plant growth in rice and contributes to salt stress tolerance by promoting the accumulation of abscisic acid (ABA)—a phytohormone that, among its numerous roles, plays a key role in abiotic stress resistance by regulating stomatal conductance, thereby impacting rates of water loss and carbon gain (Zhu et al., 2015). Two other rice MYBs (*OsMYB6* and *OsMYB48-1)* were shown to be involved in abiotic stress responses. Overexpression increased drought and salinity stress tolerance in rice, by inducing abiotic stress-responsive genes and promoting ABA signaling genes, respectively (Xiong et al., 2014; Tang et al., 2019).

Transcriptomic studies can be used to detect salinity responses in different crops. For example, in salinity-tolerant wild tomato (*Solanum chilense*) salt tolerance was shown to be dependent on differentially regulated genes related to: hormone signaling, Ca^2+^ signaling, ROS scavenging, and transcriptional regulation (Kashyap et al., 2020). Additionally, a comparative analysis on salt-stressed seedlings of domesticated tomato (*S. lycopersicum*) when cyclic guanosine monophosphate (c-GMP) was applied—a secondary messenger molecule involved in the salt stress response—showed improved plant osmotic adjustment, reduced non-stomatal water loss, and enhanced antioxidant defense pathways (Zhu et al., 2022). This improved salt tolerance coincided with the differential expression of 140 MYB-TF, suggesting that this transcription factor family could be involved in salinity responses (Zhu et al., 2022).

In rice, another connection between salinity and the MYB-TF family was observed: When growing a salinity-resistant and salinity-susceptible rice cultivar in saline conditions, transcriptomic analysis revealed two putative MYB60 transcription factors to be upregulated in the tolerant cultivar (Formentin et al., 2018). Interestingly, in *Arabidopsis* and grape (*Vitis vinifera*), these MYB60 TFs play a role in stomatal regulation (Cominelli et al., 2005; Galbiati et al., 2011). Similarly, when comparing drought-tolerant, salinity-tolerant, and susceptible rice cultivars, MYB-TFs were differentially expressed in the salinity-tolerant cultivar compared with the other two cultivars (Shankar et al., 2016). These examples demonstrate the value of transcriptomic approaches to identify important TFs. However, a targeted approach could be better suitable for known genes.

Overexpression of other MYB-TFs reduced various stresses: salt, drought, and cold stress in tomato and apple (*Malus domestica*) (Cao et al., 2013); salt and pathogen resistance in *Arabidopsis* (Shen et al., 2017); heat stress in rice (El-kereamy et al., 2012); and drought stress in cotton (*Gossypium barbadense*) (Chen et al., 2014). Similarly, transgenic maize (*Zea mays*) showed enhanced heat and drought tolerance, when *OsMYB55* was overexpressed (Casaretto et al., 2016). Another example is *SlMYB102*, an R2R3-MYB in tomato; when it was overexpressed, ROS scavenging enzyme activities and antioxidant content increased, reducing ROS content, along with increased Na^+^/K^+^ homeostasis (Zhang et al., 2020). Whereas ectopic expression of a wheat MYB (*TaMYB73*0) in *Arabidopsis* increased salinity tolerance, by improving the ionic resistance (He et al., 2012). Furthermore, *GmMYB84* was characterized as a mediator of root elongation inhibition in response to drought stress in soybean (*Glycine max*) (Wang et al., 2017), whereas silencing *AtMYB60* resulted in increased drought tolerance by constitutively reducing stomatal opening in *Arabidopsis* (Cominelli et al., 2005). The variety of species studied and the close relationship to specific functions opens new possibilities for understanding the diverse roles of MYB-TFs in various abiotic stress responses.

There is a close relationship between R2R3-MYB sequences and their function, even between different species (Allan and Espley, 2018). Phylogenetic trees can be used to discover new functions of different R2R3-MYBs, through homology in the aminoacidic sequences (Stracke et al., 2001; Dubos et al., 2010; Du et al., 2012; Hou et al., 2014; Pucker et al., 2020). For example, *CaMYB101* represses anthocyanin biosynthesis in sweet pepper (*Capsicum annuum*) and is closely related to petunia *PhMYB27*, which has the same function (Liu et al., 2021). The functional redundancy is very well illustrated by the regulation of anthocyanin biosynthesis. In *Arabidopsis*, petunia, and maize, close R2R3-MYB homologs PAP1 (AtMYB75), AN2, and C1 respectively were shown to regulate the anthocyanin pathway (Koes et al., 2005). The same was shown in other species i.e., apple, litchi (*Litchi chinensis*), and grape (Chagné et al., 2007; Cavallini et al., 2014; Lai et al., 2019). Another example is *AtMYB4*, a MYB repressor of the phenylpropanoid pathway in *Arabidopsis*. *PhMYB4* and *FaMYB1* are homolog genes of *AtMYB4* that fine-tune volatile production by controlling flavonoids in petunia and anthocyanins in strawberries (*Fragaria × ananasa*) by repressing the same gene in the phenylpropanoid pathway in both species (Jin et al., 2000; Aharoni et al., 2001; Colquhoun et al., 2011). In banana (*Musa acuminata*) fruits, *MaMYB3* acts as a repressor in the modulation of starch degradation; when overexpressing *MaMYB3* in transgenic tomatoes, this MYB prevented normal ripening of tomato fruits (Fan et al., 2018). In this context, we can take advantage of R2R3-MYBs with known functions related to abiotic stress resistance as candidates to test in other species and investigate if their function is conserved.

## Petunia as a model to study salinity

Petunia, together with *Arabidopsis* and maize, is among the first plant species where many different R2R3-MYBs with different functions were identified (Koes et al., 2005). Petunia belongs to the *Solanaceae* family, and is a perfect link between lab and crop models due to its close relation with common crops such as tomato, sweet pepper, potato (*Solanum tuberosum*), and eggplant (*Solanum melongena*) (Vandenbussche et al., 2016). Furthermore, petunia flowers have several known quality traits—e.g., fragrance, color, morphological patterns—that are regulated by R2R3-MYBs (Santos and Handro, 1983; Verdonk et al., 2005; Quattrocchio et al., 2006; Baumann et al., 2007; Hoballah et al., 2007). Similarly agronomic and horticultural traits—such as abiotic resistance—are expected to be regulated by MYB-TFs as well (Wang et al., 2020; Hussain et al., 2021). Nevertheless, petunia contains >100 hypothetical R2R3-MYBs that remain uncharacterized. Hence, we propose to identify homologous genes and new functions of different R2R3-MYBs, which could be used later in genome-wide association studies or functional characterization studies. Such studies should emulate the harsh conditions that field crops often grow under—such as salinity, drought, high light, or heat—and elucidate the R2R3-MYBs related to a given stress response.

**Figure 1:**
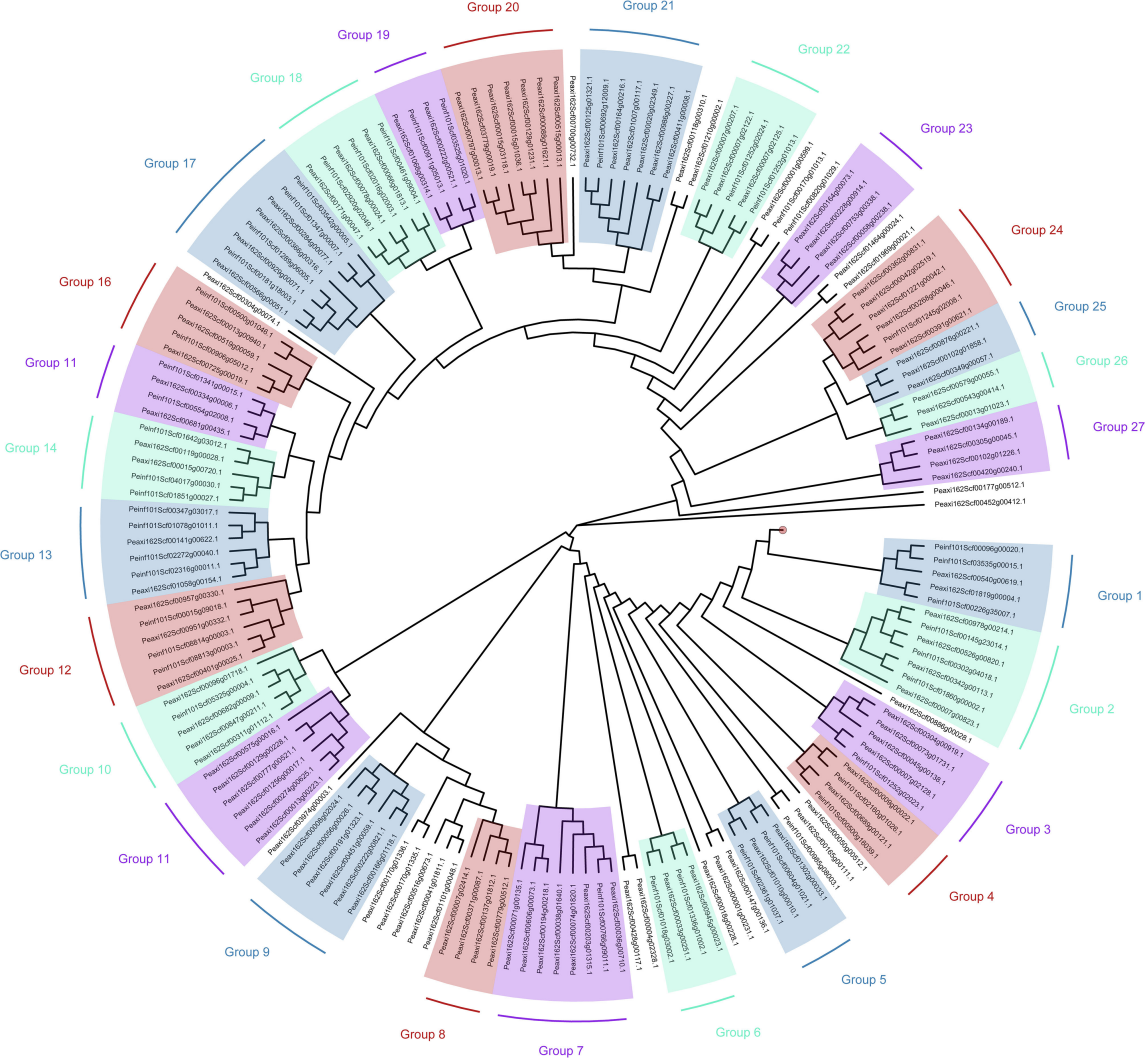
Phylogenetic tree with R2R3-MYBs based on aminoacidic sequences of *Petunia axilallis* and *Petunia inflata*. (1) The sequences of the MYB-TF family were retrieved from the Plant transcription factor database (Jin et al., 2017). (2) Using the Levenshtein distance method in R, the dissimilarity between sequences was measured (Pagès et al., 2023). (3) The phylogenetic tree was then built using the BioNJ algorithm (Gascuel, 1997), and (4) visually illustrating the relationships among the sequences using (Yu et al., 2017). The red circle between Group 27 and Group 1 represents 23 individual clades, each with single sequences, that were omitted from this tree.

We made a phylogenetic tree (**Figure 1**) to group the two openly available genome sequences of petunia: *Petunia axillaris and Petunia inflata* (Jin et al., 2017). This tree can be used to predict complete groups with potential homologous genes related to salinity resistance. R2R3-MYBs can be visualized with 27 groups of R2R3-MYBs with homologous sequences. With this strategy, we aim to have these groups as a foundation for future studies and help characterize stress tolerance in different species.

We constructed the phylogenetic tree with information from both *Petunia axilaris* and *Petunia inflata* because of gene redundancies during their evolution (Bombarely et al., 2016). Gene redundancies are important because there are examples of paralogs and diversification in petunia: for example *AN2* and *AN4* are genes that code for R2R3-MYBs in charge of flower coloration. These are expressed in different locations in flower tissues and flower developmental stages, producing a clear difference in anthocyanin expression between the two wild parental lines (Bombarely et al., 2016, Supplementary note 7). To confirm the previous duplication and potential search of salinity related R2R3-MYB, we align previously mentioned MYB-TFs: *OsMYB55, OsMYB91*, and *AtMYB60*. For *OsMYB55*, group 10 (Peaxi162Scf00311g01112.1, Peaxi162Scf00096g01718.1, Peaxi162Scf00682g00009.1, and Peaxi162Scf00847g00211.1, including the sequence for *Petunia inflata* :Peinf101Scf05325g00004.1) showed the homolog sequence in the same clade. The homolog sequence, on the other hand, for *OsMYB91* and *AtMYB60* were part of group 11. This example suggests that groups 10 and 11 in this tree could be related to abiotic stress resistance in petunia, and it may help future studies to begin with these groups first. With this strategy, it will be possible to transfer this knowledge from petunia to other species.

Petunia presents a high tolerance to a number of abiotic stresses. Petunia is more salinity tolerant when compared with tomato, eggplant, or potato, which are respectively moderately salt-tolerant, moderately salt-sensitive, and sensitive to salinity (Fornes et al., 2007; Ünlükara et al., 2010; Villarino and Mattson, 2011; Charfeddine et al., 2019; Moles et al., 2019; Parkash and Singh, 2020; Altaf et al., 2023). Under stressful conditions such as high light intensity, petunia modulates vegetative anthocyanin and volatile production as a coping mechanism (Albert et al., 2009; Colquhoun et al., 2013). Nevertheless, even when petunia presents tolerance to abiotic stresses, the current trend is to study the final phenotype—physiological and metabolomics responses—but understanding transcriptomic regulation could be a more direct way to improve resistance when breeding for new varieties. Some transcriptomics studies have been conducted to identify the different pathways involved in salinity resistance mechanisms on petunia under salinity stress (Villarino et al., 2014). For example, salinity and drought resistance are partially achieved by modulating ion transport inside the vacuoles (Xu et al., 2009; Asif et al., 2011; Banjara et al., 2012). In these studies, the overexpression of *AtNHX1*—a vacuolar Na^+^/H^+^ antiporter gene—significantly enhanced the resistance to salinity and drought stresses. The transgenic expression of *FvMYB24* in *Arabidopsis* enhanced the salinity tolerance of transgenic plants, and among other genes *AtNHX1* was upregulated (Wang S. et al., 2021). However, the connection between those studies and intermediary regulatory pathways remains uncharacterized.

To summarize, we argue that salinity is a problem affecting commercially important crops worldwide, and that studying R2R3-MYBs in petunia could help to address this issue. Petunia, as a member of the *Solanaceae* family, is a suitable link between lab and crop models, and is highly salinity tolerant. We suggest the use of petunia as a model plant to study salinity resistance and to identify the R2R3-MYB regulators in response to salinity. We picked R2R3-MYB, because they are known to be related with different developmental pathways, specialized metabolisms pathways, and stress responses pathways. The most important aspect is that the function of those MYB-TFs is usually maintained between different plant species, making the extrapolation of these discoveries to other crops feasible. We proposed to functionally characterize these MYB-TFs by the use of homology and phylogeny. Here, we present an example based on *Petunia axillaris* and *Petunia inflata*, and suggest that it should be possible to extrapolate this idea to other crops with known sequences.

## Data availability statement

The original contributions presented in the study are included in the article/supplementary files, further inquiries can be directed to the corresponding author.

## Author contributions

BZ: Conceptualization, Data curation, Formal Analysis, Funding acquisition, Investigation, Methodology, Software, Validation, Visualization, Writing – original draft, Writing – review and editing. LM: Conceptualization, Project administration, Supervision, Writing – original draft, Writing – review and editing, Funding acquisition, Investigation. EK: Conceptualization, Writing – original draft, Writing – review and editing, Investigation, Project administration, Supervision. JV: Conceptualization, Investigation, Project administration, Supervision, Writing – original draft, Writing – review and editing, Funding acquisition.
